# General Notes

**Published:** 1893-12

**Authors:** 


### GENERAL NOTES.

Walter Baker & Co., Dorchester, Mass., have received from the Judges of the World's Columbian Exposition one of the highest awards on each of the following named articles contained in their exhibit: Breakfast Cocoa, No. 1 Chocolate, German Sweet Chocolate, Vanilla Chocolate, Cocoa Butter.

The Judges state in their report that these products are characterized by excellent flavor, purity of material employed, and uniform even composition, indicating great care in point of mechanical preparation.

There is one live American who has the credit over his European friends of bringing to the surface of science, a long looked-for event, but only recently accomplished. It is that of applying the famous Australian Electro Pill remedy in a similar form to electricity for certain diseases. It seems to act upon the nervous system similar to the electro current, in which kidney, liver and stomach trouble and sympathetic diseases yield at once to its subtile healing powers. The manufacturer of this remedy has kindly offered to mail each of our readers seven days treatment free of charge, if you kindly name HALL'S JOURNAL OF HEALTH, and address Dr. E. J. Worst, Ashland, O.

The human body is composed of a grand aggregation of animal molecules that prey upon each other. Fermentation of food in the stomach is, and in fact nearly all bodily disorders are caused by microbes warring upon each other; this is the microbean theory of disease. All high class animal and vegetable foods contain thousands of such microbes; only low order of animal food, such as clams or oysters, being free from them. So in all stomach troubles, use Burnham's Clam Bouillon. It goes back to first principles. All druggists and grocers.

That much dreaded disease La Grippe having again put in its appearance, gives every one a word of warning to prepare for it. Herba Vita is the greatest cure yet discovered for it.

The following testimonial speaks for itself: "Herba Vita Remedy Co., New York city. Those here who have tried your Herba Vita tea say it is a good thing and especially for La Grippe. W. W. STANTON, Druggist, Quakertown, Ind."

If you are a sufferer of Piles, or desire to stop the filthy habit of tobacco chewing, you should not hesitate to put yourself in communication with Dr. J. W. Chiles, Dixon, Ill.

Prof. G. Birkholz, of Chicago, who has an office in New York, at 2 West Fourteenth street, pays particular attention to the subject of bald heads and their cure.

Look for his advertisement in this issue of the JOURNAL.

